# Supplementary material for: Trends in heart failure-related cardiovascular mortality in rural versus urban United States counties, 2011–2018: A cross-sectional study
Source: PLoS One. 2021 Mar 3;16(3):e0246813. doi: 10.1371/journal.pone.0246813 (PMC7928489; doi:10.1371/journal.pone.0246813)
Supplement: S1 Table — *Most recent publicly available versions of the respective data sources. (DOCX) [file pone.0246813.s002.docx]

**S1 Table**. County-level factor data sources and years used in primary and sensitivity analyses.

| **County-level factor** | **Source** | **File** | **Year used in primary analysis** | **Year used in sensitivity analysis*** |
| --- | --- | --- | --- | --- |
| **Demographic characteristics of residents, %** |  |  |  |  |
| Age > 65 years | U.S. Census Bureau | Population and Housing Unit Estimates | 2011 | 2018 |
| Female | U.S. Census Bureau | Population and Housing Unit Estimates | 2011 | 2018 |
| Non-Hispanic Black | U.S. Census Bureau | Population and Housing Unit Estimates | 2011 | 2018 |
| Hispanic | U.S. Census Bureau | Population and Housing Unit Estimates | 2011 | 2018 |
| **Socioeconomic characteristics of residents** |  |  |  |  |
| In poverty, % | U.S. Census Bureau | Small Area Income and Poverty Estimates Program | 2011 | 2018 |
| Unemployed, % | U.S. Bureau of Labor Statistics | Local Area Unemployment Statistics | 2011 | 2018 |
| Uninsured residents age 18-64, % | U.S. Census Bureau | Small Area Health Insurance Estimates Program | 2011 | 2017 |
| Median household income, $ | U.S. Census Bureau | Small Area Income and Poverty Estimates Program | 2011 | 2018 |
| **Clinical characteristics of residents, %** |  |  |  |  |
| With diabetes | Centers for Disease Control and Prevention | Behavioral Risk Factor Surveillance System | 2011 | 2016 |
| With obesity | Centers for Disease Control and Prevention | Behavioral Risk Factor Surveillance System | 2011 | 2016 |
| **Clinicians per 100,000 residents** |  |  |  |  |
| Primary care physicians | Health Resources and Services Administration | Area Health Resources Files | 2011 | 2017 |
| Cardiologists | Health Resources and Services Administration | Area Health Resources Files | 2010 | 2017 |

*Most recent publicly available versions of the respective data source
