# Supplementary material for: Trends in heart failure-related cardiovascular mortality in rural versus urban United States counties, 2011–2018: A cross-sectional study
Source: PLoS One. 2021 Mar 3;16(3):e0246813. doi: 10.1371/journal.pone.0246813 (PMC7928489; doi:10.1371/journal.pone.0246813)
Supplement: S2 Table — HF = heart failure; y = years. *Rural-urban status grouped based on the 2013 NCHS Urban-Rural Classification Scheme for Counties. †Expressed as number of deaths per 100,000 residents. (DOCX) [file pone.0246813.s003.docx]

**S2 Table.** Crude number of deaths, crude death rate, and percent of deaths for heart failure-related mortality stratified by urban-rural classification and race-sex groups, Center for Disease Control and Prevention Wide-Ranging Online Data for Epidemiologic Research 2011-2018.

| **Urban-rural classification** | **Overall** | | | **Age 35-64 y** | | | **Age 65-84 y** | | |
| --- | --- | --- | --- | --- | --- | --- | --- | --- | --- |
|  | **Deaths** | **% total HF-related deaths** | **Crude rate**† | **Deaths** | **% total HF-related deaths** | **Crude rate**† | **Deaths** | **% total HF-related deaths** | **Crude rate**† |
| **Rural*** |  |  |  |  |  |  |  |  |  |
| Overall | 162,314 | 21.9 | 83.3 | 28,229 | 20.9 | 20.4 | 134,085 | 22.1 | 237.1 |
| Black women | 6,974 | 0.9 | 92.0 | 2,105 | 1.6 | 36.7 | 4,869 | 0.8 | 263.7 |
| White Women | 62,707 | 8.4 | 68.9 | 7,959 | 5.9 | 12.6 | 54,748 | 9.0 | 194.9 |
| Black men | 8,051 | 1.1 | 108.2 | 3,223 | 2.4 | 53.3 | 4,828 | 0.8 | 345.5 |
| White men | 84,582 | 11.4 | 95.3 | 14,942 | 11.1 | 23.5 | 69,640 | 11.5 | 276.2 |
| **Urban*** |  |  |  |  |  |  |  |  |  |
| Overall | 580,305 | 78.1 | 56.2 | 106,740 | 79.1 | 13.7 | 473,565 | 77.9 | 187.7 |
| Black women | 44,420 | 6.0 | 55.8 | 13,045 | 9.7 | 20.6 | 31,375 | 5.2 | 192.8 |
| White Women | 204,730 | 27.6 | 44.9 | 24,329 | 18.0 | 7.3 | 1804,01 | 29.7 | 147.9 |
| Black men | 50,592 | 6.8 | 77.0 | 19,942 | 14.8 | 36.7 | 30,650 | 5.0 | 268.6 |
| White men | 280,563 | 37.8 | 65.0 | 49,424 | 36.6 | 15.0 | 231,139 | 38.0 | 225.0 |

HF = heart failure; y = years

* Rural-urban status grouped based on the 2013 NCHS Urban-Rural Classification Scheme for Counties.

† Expressed as number of deaths per 100,000 residents
