# Supplementary material for: Trends in heart failure-related cardiovascular mortality in rural versus urban United States counties, 2011–2018: A cross-sectional study
Source: PLoS One. 2021 Mar 3;16(3):e0246813. doi: 10.1371/journal.pone.0246813 (PMC7928489; doi:10.1371/journal.pone.0246813)
Supplement: S3 Table — No. = number; Y = years. *Rural-urban status grouped based on the 2013 NCHS Urban-Rural Classification Scheme for Counties. †Percent of residents over age 65 years, percent of female residents, percent of non-Hispanic Black residents, and percent of Hispanic residents according to the US Census Bureau 2011 Population and Housing Unit Estimates. ‡Percent of residents in poverty, percent of residents unemployed, and median household income according to the US Census Bureau 2018 Small Area Income and Poverty Estimates Program, and percent of residents uninsured (age 18–64) according to 2011 Small Area Health Insurance Estimates Program. §Percent of residents with diabetes and percent of residents with obesity from the 2011 Behavioral Risk Factor Surveillance System. | | Primary care physicians and number of cardiologists per 100,000 residents according to the Health Resources and Services Area Health Resources File 2011 and 2010 statistics, respectively. (DOCX) [file pone.0246813.s004.docx]

**S3 Table.** Mean county-level characteristics stratified by age and rurality, 2011.

|  | **Age 35-64 y** |  | **Age 65-84 y** |  |
| --- | --- | --- | --- | --- |
| **Characteristic** | **Urban* (n=818)** | **Rural* (n=505)** | **Urban***  **(n=1,130)** | **Rural***  **(n=1,541)** |
| **County population, No. (SD)** | 916,685  (1,559,985) | 140,132  (79,010) | 223,102  (405,139) | 35,133  (26,408) |
| **Demographic characteristics of residents**†**, % (SD)** |  |  |  |  |
| Age > 65 years | 13.7 (3.6) | 16.0 (2.9) | 14.0 (3.5) | 17.0 (3.6) |
| Female | 50.8 (1.1) | 50.4 (1.9) | 50.6 (1.5) | 49.9 (2.3) |
| Non-Hispanic Black | 13.4 (14.0) | 15.0 (18.4) | 11.7 (13.6) | 9.6 (15.9) |
| Hispanic | 10.5 (13.1) | 6.5 (9.7) | 9.1 (12.1) | 7.5 (13.2) |
| **Socioeconomic characteristics of residents**‡ |  |  |  |  |
| In poverty, % (SD) | 15.7 (5.3) | 20.9 (6.1) | 15.3 (5.5) | 19.0 (6.5) |
| Unemployed, % (SD) | 8.9 (2.4) | 10.3 (2.6) | 8.7 (2.4) | 9.3 (3.0) |
| Uninsured residents age 18-64, % (SD) | 20.3 (6.3) | 23.5 (5.5) | 20.1 (6.3) | 22.5 (6.3) |
| Median household income, $ (SD) | 50,272  (12,270) | 37,924  (6,780) | 50,124 (12,604) | 39,470 (7,528) |
| **Clinical characteristics of residents**§**, %** (SD) |  |  |  |  |
| With diabetes | 10.4 (2.1) | 12.3 (2.2) | 10.5 (2.2) | 11.5 (2.3) |
| With obesity | 29.5 (4.6) | 32.8 (4.1) | 29.8 (4.4) | 31.6 (4.2) |
| **Clinicians per 100,000 residents**\| \|, **No. (SD)** |  |  |  |  |
| Primary care physicians | 66.6 (31.1) | 51.7 (22.7) | 60.8 (36.2) | 50.4 (27.2) |
| Cardiologists | 5.9 (5.8) | 2.3 (3.6) | 4.9 (7.2) | 1.2 (2.9) |

No. = number; Y = years

* Rural-urban status grouped based on the 2013 NCHS Urban-Rural Classification Scheme for Counties

† Percent of residents over age 65 years, percent of female residents, percent of non-Hispanic Black residents, and percent of Hispanic residents according to the US Census Bureau 2011 Population and Housing Unit Estimates.

‡ Percent of residents in poverty, percent of residents unemployed, and median household income according to the US Census Bureau 2018 Small Area Income and Poverty Estimates Program, and percent of residents uninsured (age 18-64) according to 2011 Small Area Health Insurance Estimates Program.

§ Percent of residents with diabetes and percent of residents with obesity from the 2011 Behavioral Risk Factor Surveillance System.

| | Primary care physicians and number of cardiologists per 100,000 residents according to the Health Resources and Services Area Health Resources File 2011 and 2010 statistics, respectively.
