# Supplementary material for: Trends in heart failure-related cardiovascular mortality in rural versus urban United States counties, 2011–2018: A cross-sectional study
Source: PLoS One. 2021 Mar 3;16(3):e0246813. doi: 10.1371/journal.pone.0246813 (PMC7928489; doi:10.1371/journal.pone.0246813)
Supplement: S4 Table — IRR = incidence rate ratio; y = years. *Rural-urban status grouped based on the 2013 NCHS Urban-Rural Classification Scheme for Counties. †Adjusted demographic factors including percent of residents over age 65 years, percent of female residents, percent of non-Hispanic Black residents, and percent of Hispanic residents according to the US Census Bureau 2018 Population and Housing Unit Estimates. ‡Adjusted for socioeconomic factors including percent of residents in poverty, percent of residents unemployed, percent of residents uninsured age 18–64, and median household income according to the US Census Bureau 2018 Small Area Income and Poverty Estimates Program and 2017 Small Area Health Insurance Estimates Program. §Adjusted for clinical characteristics of residents including percent of residents with diabetes and percent of residents with obesity from the 2016 Behavioral Risk Factor Surveillance System. | |Adjusted for clinician density including number of primary care physicians and number of cardiologists per 100,000 residents according to the Health Resources and Services Area Health Resources File 2017 statistics. ¶Adjusted for all covariates in models 2–5. (DOCX) [file pone.0246813.s005.docx]

**S4 Table.** Multivariable negative binomial regression modeling of latest aggregate county-level factors contributing to excess heart failure-related mortality in rural* counties, Center for Disease Control and Prevention Wide-Ranging Online Data for Epidemiologic Research 2011-2018.

|  | **Age 35-64 y** | **Age 65-84 y** |
| --- | --- | --- |
| **Model** | **IRR (95% CI)** | **IRR (95% CI)** |
| Model 1: Unadjusted | 1.67 (1.57-1.78) | 1.16 (1.13-1.18) |
| Model 2: Demographic† | 1.60 (1.52-1.70) | 1.20 (1.18-1.23) |
| Model 3: Socioeconomic‡ | 1.07 (1.01-1.12) | 1.01 (0.99-1.03) |
| Model 4: Clinical§ | 1.33 (1.26-1.41) | 1.10 (1.08-1.13) |
| Model 5: Physician density\| \| | 1.53 (1.44-1.63) | 1.13 (1.10-1.15) |
| Model 6: Fully-adjusted¶ | 1.10 (1.05-1.16) | 1.05 (1.02-1.07) |

IRR = incidence rate ratio; y = years

* Rural-urban status grouped based on the 2013 NCHS Urban-Rural Classification Scheme for Counties.

† Adjusted demographic factors including percent of residents over age 65 years, percent of female residents, percent of non-Hispanic Black residents, and percent of Hispanic residents according to the US Census Bureau 2018 Population and Housing Unit Estimates.

‡ Adjusted for socioeconomic factors including percent of residents in poverty, percent of residents unemployed, percent of residents uninsured age 18-64, and median household income according to the US Census Bureau 2018 Small Area Income and Poverty Estimates Program and 2017 Small Area Health Insurance Estimates Program.

§ Adjusted for clinical characteristics of residents including percent of residents with diabetes and percent of residents with obesity from the 2016 Behavioral Risk Factor Surveillance System.

| | Adjusted for clinician density including number of primary care physicians and number of cardiologists per 100,000 residents according to the Health Resources and Services Area Health Resources File 2017 statistics.

¶ Adjusted for all covariates in models 2-5.
