# Supplementary material for: Trends in heart failure-related cardiovascular mortality in rural versus urban United States counties, 2011–2018: A cross-sectional study
Source: PLoS One. 2021 Mar 3;16(3):e0246813. doi: 10.1371/journal.pone.0246813 (PMC7928489; doi:10.1371/journal.pone.0246813)
Supplement: S5 Table — IRRs for HF-related mortality between quintiles of individual county-level factors in fully-adjusted model relative to lowest quintile. Results are representative of four fully-adjusted negative binomial regression models for age (35-64y and 65-84y) and rural-urban subgroups. Data sources as in Fig 3. HF = heart failure; IRR = incidence rate ratio; y = year. (DOCX) [file pone.0246813.s006.docx]

**S5 Table.** Associations between county-level factors and age-adjusted heart failure-related mortality rates stratified by rural-urban status and age, Center for Disease Control and Prevention Wide-Ranging Online Data for Epidemiologic Research 2011-2018.

|  | **IRR (95% CI)** | | | | | | |
| --- | --- | --- | --- | --- | --- | --- | --- |
|  | **Age 35-64 y** | |  | | **Age 65-84 y** | |  |
| **Characteristic** | **Urban**  **(n=818)** | **Rural**  **(n=505)** | | **Urban**  **(n=1,130)** | | **Rural**  **(n=1,541)** | |
| **Demographic characteristics of residents** |  |  | |  | |  | |
| Age > 65 years |  |  | |  | |  | |
| Quintile 1 | Ref. | Ref. | | Ref. | | Ref. | |
| Quintile 2 | 1.00 (0.93, 1.07) | 0.90 (0.79, 1.02) | | 0.98 (0.94, 1.01) | | 0.97 (0.92, 1.03) | |
| Quintile 3 | 0.96 (0.88, 1.05) | 0.86 (0.75, 0.98) | | 0.94 (0.90, 0.98) | | 0.96 (0.91, 1.01) | |
| Quintile 4 | 0.99 (0.87, 1.12) | 0.91 (0.79, 1.04) | | 0.93 (0.89, 0.98) | | 0.92 (0.87, 0.97) | |
| Quintile 5 | 0.85 (0.74, 1.00) | 0.90 (0.76, 1.08) | | 0.76 (0.71, 0.82) | | 0.87 (0.82, 0.92) | |
| Female |  |  | |  | |  | |
| Quintile 1 | Ref. | Ref. | | Ref. | | Ref. | |
| Quintile 2 | 1.00 (0.88, 1.13) | 1.17 (1.02, 1.34) | | 1.04 (0.99, 1.10) | | 1.02 (0.98, 1.07) | |
| Quintile 3 | 1.07 (0.95, 1.20) | 1.04 (0.93, 1.17) | | 1.07 (1.01, 1.12) | | 1.02 (0.98, 1.06) | |
| Quintile 4 | 0.94 (0.84, 1.05) | 1.08 (0.97, 1.21) | | 1.03 (0.98, 1.09) | | 1.02 (0.98, 1.06) | |
| Quintile 5 | 1.01 (0.90, 1.13) | 1.17 (1.05, 1.30) | | 1.05 (0.99, 1.11) | | 1.03 (0.99, 1.08) | |
| Non-Hispanic Black |  |  | |  | |  | |
| Quintile 1 | Ref. | Ref. | | Ref. | | Ref. | |
| Quintile 2 | 1.23 (0.97, 1.57) | 1.01 (0.86, 1.20) | | 1.04 (0.97, 1.11) | | 0.98 (0.95, 1.03) | |
| Quintile 3 | 1.26 (1.00, 1.58) | 0.96 (0.82, 1.13) | | 1.03 (0.97, 1.10) | | 1.03 (0.99, 1.07) | |
| Quintile 4 | 1.31 (1.04, 1.65) | 1.07 (0.90, 1.26) | | 0.99 (0.93, 1.06) | | 1.07 (1.02, 1.13) | |
| Quintile 5 | 1.52 (1.20, 1.92) | 1.17 (0.98, 1.39) | | 0.97 (0.91, 1.04) | | 1.04 (0.99, 1.10) | |
| Hispanic |  |  | |  | |  | |
| Quintile 1 | Ref. | Ref. | | Ref. | | Ref. | |
| Quintile 2 | 0.99 (0.87, 1.13) | 0.90 (0.82, 1.00) | | 1.03 (0.98, 1.08) | | 0.95 (0.91, 0.98) | |
| Quintile 3 | 0.96 (0.84, 1.09) | 0.87 (0.78, 0.97) | | 0.97 (0.93, 1.02) | | 0.94 (0.91, 0.98) | |
| Quintile 4 | 0.92 (0.81, 1.05) | 0.87 (0.78, 0.97) | | 0.98 (0.93, 1.03) | | 0.96 (0.91, 1.00) | |
| Quintile 5 | 0.86 (0.75, 1.01) | 0.91 (0.79, 1.06) | | 0.92 (0.87, 0.98) | | 0.93 (0.88, 0.99) | |
| **Socioeconomic characteristics of residents** |  |  | |  | |  | |
| In poverty |  |  | |  | |  | |
| Quintile 1 | Ref. | Ref. | | Ref. | | Ref. | |
| Quintile 2 | 1.07 (0.98, 1.18) | 0.94 (0.73, 1.21) | | 1.02 (0.98, 1.07) | | 0.97 (0.92, 1.03) | |
| Quintile 3 | 1.09 (0.97, 1.23) | 1.13 (0.85, 1.50) | | 1.04 (0.98, 1.10) | | 0.97 (0.91, 1.04) | |
| Quintile 4 | 1.22 (1.06, 1.40) | 1.12 (0.84, 1.50) | | 1.08 (1.01, 1.15) | | 0.94 (0.87, 1.02) | |
| Quintile 5 | 1.36 (1.14, 1.62) | 1.23 (0.90, 1.67) | | 1.09 (1.00, 1.19) | | 0.95 (0.87, 1.04) | |
| Unemployed |  |  | |  | |  | |
| Quintile 1 | Ref. | Ref. | | Ref. | | Ref. | |
| Quintile 2 | 0.93 (0.84, 1.03) | 0.79 (0.66, 0.96) | | 1.02 (0.97, 1.06) | | 0.94 (0.90, 0.99) | |
| Quintile 3 | 0.96 (0.87, 1.07) | 0.75 (0.62, 0.89) | | 1.03 (0.98, 1.08) | | 0.91 (0.87, 0.96) | |
| Quintile 4 | 0.92 (0.82, 1.03) | 0.74 (0.62, 0.89) | | 0.98 (0.93, 1.03) | | 0.91 (0.87, 0.96) | |
| Quintile 5 | 0.96 (0.85, 1.09) | 0.76 (0.63, 0.91) | | 1.01 (0.95, 1.07) | | 0.91 (0.86, 0.96) | |
| Uninsured residents age 18-64 |  |  | |  | |  | |
| Quintile 1 | Ref. | Ref. | | Ref. | | Ref. | |
| Quintile 2 | 1.28 (1.17, 1.40) | 1.00 (0.85, 1.17) | | 1.06 (1.02, 1.10) | | 1.04 (0.99, 1.09) | |
| Quintile 3 | 1.41 (1.28, 1.55) | 1.13 (0.96, 1.34) | | 1.10 (1.05, 1.15) | | 1.08 (1.02, 1.14) | |
| Quintile 4 | 1.45 (1.30, 1.61) | 1.22 (1.02, 1.44) | | 1.11 (1.05, 1.16) | | 1.12 (1.06, 1.18) | |
| Quintile 5 | 1.51 (1.33, 1.71) | 1.22 (1.01, 1.48) | | 1.11 (1.04, 1.18) | | 1.12 (1.05, 1.20) | |
| Median household income |  |  | |  | |  | |
| Quintile 1 | Ref. | Ref. | | Ref. | | Ref. | |
| Quintile 2 | 0.88 (0.78, 1.00) | 0.84 (0.76, 0.94) | | 1.01 (0.94, 1.08) | | 0.92 (0.88, 0.96) | |
| Quintile 3 | 0.86 (0.74, 0.98) | 0.75 (0.65, 0.86) | | 1.00 (0.94, 1.08) | | 0.89 (0.84, 0.95) | |
| Quintile 4 | 0.81 (0.69, 0.95) | 0.72 (0.58, 0.89) | | 0.98 (0.90, 1.06) | | 0.83 (0.77, 0.89) | |
| Quintile 5 | 0.71 (0.60, 0.86) | 0.56 (0.40, 0.79) | | 0.94 (0.86, 1.03) | | 0.76 (0.69, 0.84) | |
| **Clinical characteristics of residents** |  |  | |  | |  | |
| With diabetes |  |  | |  | |  | |
| Quintile 1 | Ref. | Ref. | | Ref. | | Ref. | |
| Quintile 2 | 1.09 (0.99, 1.19) | 0.95 (0.85, 1.17) | | 1.04 (1.00, 1.08) | | 1.07 (1.02, 1.12) | |
| Quintile 3 | 1.21 (1.09, 1.34) | 1.07 (0.89, 1.29) | | 1.10 (1.05, 1.15) | | 1.10 (1.04, 1.16) | |
| Quintile 4 | 1.25 (1.11, 1.42) | 1.10 (0.91, 1.33) | | 1.10 (1.03, 1.16) | | 1.10 (1.04, 1.17) | |
| Quintile 5 | 1.21 (1.04, 1.40) | 1.05 (0.85, 1.28) | | 1.11 (1.03, 1.20) | | 1.07 (1.00, 1.15) | |
| With obesity |  |  | |  | |  | |
| Quintile 1 | Ref. | Ref. | | Ref. | | Ref. | |
| Quintile 2 | 1.16 (1.06, 1.27) | 1.05 (0.88, 1.25) | | 1.13 (1.08, 1.17) | | 1.08 (1.03, 1.14) | |
| Quintile 3 | 1.19 (1.08, 1.31) | 1.13 (0.95, 1.34) | | 1.15 (1.10, 1.21) | | 1.10 (1.04, 1.15) | |
| Quintile 4 | 1.29 (1.15, 1.43) | 1.10 (0.92, 1.30) | | 1.20 (1.14, 1.26) | | 1.14 (1.08, 1.20) | |
| Quintile 5 | 1.34 (1.19, 1.51) | 1.22 (1.02, 1.46) | | 1.21 (1.14, 1.28) | | 1.18 (1.11, 1.25) | |
| **Clinicians per 100,000 residents** |  |  | |  | |  | |
| Primary care physicians |  |  | |  | |  | |
| Quintile 1 | Ref. | Ref. | | Ref. | | Ref. | |
| Quintile 2 | 0.89 (0.80, 0.99) | 0.86 (0.76, 0.98) | | 0.96 (0.92, 1.01) | | 1.00 (0.96, 1.04) | |
| Quintile 3 | 0.93 (0.83, 1.05) | 0.82 (0.73, 0.93) | | 0.97 (0.93, 1.02) | | 0.98 (0.94, 1.02) | |
| Quintile 4 | 0.88 (0.78, 1.00) | 0.80 (0.70, 0.92) | | 0.96 (0.92, 1.01) | | 0.94 (0.90, 0.99) | |
| Quintile 5 | 0.95 (0.83, 1.08) | 0.76 (0.65, 0.89) | | 0.98 (0.92, 1.04) | | 0.92 (0.88, 0.97) | |
| Cardiologists |  |  | |  | |  | |
| Quintile 1-3 | Ref. | Ref. | | Ref. | | Ref. | |
| Quintile 4 | 0.90 (0.82, 0.99) | 0.86 (0.79, 0.93) | | 0.98 (0.94, 1.02) | | 0.94 (0.91, 0.98) | |
| Quintile 5 | 0.87 (0.78, 0.97) | 0.88 (0.80, 0.96) | | 0.93 (0.89, 0.98) | | 0.93 (0.89, 0.97) | |

Data sources as in Fig 3. HF = heart failure; IRR = incidence rate ratio; y = year
