# Supplementary material for: Trends in heart failure-related cardiovascular mortality in rural versus urban United States counties, 2011–2018: A cross-sectional study
Source: PLoS One. 2021 Mar 3;16(3):e0246813. doi: 10.1371/journal.pone.0246813 (PMC7928489; doi:10.1371/journal.pone.0246813)
Supplement: Attachment — Submitted filename: Response to reviewers_Final.docx [file pone.0246813.s007.docx]

**Requests from the editors:**

**Comment 1: *At this stage, we ask that you include a short, non-technical Author Summary of your research to make findings accessible to a wide audience that includes both scientists and non-scientists. The Author Summary should immediately follow the Abstract in your revised manuscript. This text is subject to editorial change and should be distinct from the scientific abstract. Please see our author guidelines for more information.** 

Authors’ Response: For our PLOS ONE resubmission, we have removed this section in order to best fit the PLOS ONE manuscript preparation guidelines.

**Comment 2: *In the paper, please clarify whether the analytical approach followed here corresponded to one laid out in a prospective protocol or analysis plan? Please state this (either way) early in the Methods section.**

Authors’ Response: Thank you for this query. Yes, we used a prospective statistical analysis plan and have added this to the Methods section.

**a) If a prospective analysis plan (from your funding proposal, IRB or other ethics committee submission, study protocol, or other planning document written before analyzing the data) was used in designing the study, please include the relevant prospectively written document with your revised manuscript as a Supporting Information file to be published alongside your study, and cite it in the Methods section. A legend for this file should be included at the end of your manuscript.** 

Authors’ Response: Due to the publicly available, deidentified nature of these data, our study was exempt from review by the Northwestern University Institutional Review Board. However, the methods and analysis plan were developed prospectively by the first and senior authors (J.B.P and S.S.K.) prior to the initiation of data procurement and analysis. The final analytic plan was decided on February 11, 2020. Analysis occurred between February 12, 2020 through March 27, 2020. This has now been documented in the methods section for transparency.

**b) If no such document exists, please make sure that the Methods section transparently describes when analyses were planned, and when/why any data-driven changes to analyses took place.**

Authors’ Response: We have revised the manuscript to include the details of the prospective analysis plan described above, which now reads:

- Page 5, lines 122-124: *“A pre-specified analytic plan was finalized on February 11, 2020. Statistical analysis occurred between February 12 through March 27, 2020.”*

**c) In either case, changes in the analysis-- including those made in response to peer review comments-- should be identified as such in the Methods section of the paper, with rationale.**

Authors’ Response: No changes were made to the analytic plan after finalization on February 11^th^ and completion on March 27^th^, 2020. All changes made in response to peer review comments are outlined below.
-----------------------------------------------------------

**Comments from the reviewers:

Reviewer #1: The study by Pierce et al. demonstrated the trends in HF-related mortality in rural vs. urban US countries using CDC database. It is well designed and manuscript is well organized. There are several points to be addressed.**

Authors’ Response: We thank the reviewer for their time and appreciate the overall enthusiasm regarding our work. We have responded in detail to the reviewer’s suggestions below.

**Comment 1: page 4 lines 66-67. I could not understand why HF-related mortality has begun to increase since 2011, after I read ref 4-6. Therapies has progressed (ARNI, devices, SGLT2Is, etc) so that mortality should be decreased when it was age-adjusted (AAMR). If there is no explanation for this evidence, readers may think it is a problem of diagnosis of HF, as the authors mentioned several times over the manuscript, especially in the limitation section. This is the fatal point of this study. As the author stated, low socioeconomic / uninsured persons may easily be diagnosed as HF without detailed examination. Please explain this point.**

Authors’ Response: We thank the reviewer for raising this important point. We agree with the reviewer that there have been significant scientific advances in the medical management of heart failure such as SGLT2-inhibitors, ARNIs, and circulatory support devices. However, despite the benefits demonstrated in several high-profile clinical trials, substantial evidence demonstrates poor implementation of these life-saving therapies in routine clinical practice.^1,2^ Indeed, patients are very frequently either not treated or undertreated even with established therapies such as beta blockers, RAAS inhibitors, and MRAs whereby <1% of patients are reported to be on optimal guideline-directed medical therapy for heart failure.^1^ As a result, the advances in the treatment of heart failure are unlikely to have made significant impact on nationwide mortality rates.

Conversely, the prevalence of risk factors for heart failure-related morbidity and mortality such as diabetes and obesity have been rising at alarming rates.^3-5^ Importantly, our recent analysis of years of potential life lost (YPLL) due to heart failure has increased over the study period, suggesting that there is increasing premature mortality from heart failure.^6^ The etiology of the inflection point in heart failure mortality that occurred in 2011 is likely multifactorial, with the above trends playing important roles. We have included a description of potential contributions to increasing HF mortality in the manuscript, which now reads:

- Page 4, lines 102-105: *“As declines in the CVD mortality rate in the US have stalled, heart failure (HF)-related mortality has begun to increase [4-6] since 2011, likely as a result of increasing prevalence of risk factors for HF-related mortality [7-10] concurrent with poor implementation of guideline-directed medical therapy and novel therapeutic agents [11, 12].”*

^1^Greene SJ, Fonarow GC, DeVore AD, Sharma PP, Vaduganathan M, Albert NM, et al. Titration of medical therapy for heart failure with reduced ejection fraction. Journal of the American College of Cardiology. 2019;73(19):2365-83.

^2^Luo N, Fonarow GC, Lippmann SJ, Mi X, Heidenreich PA, Yancy CW, et al. Early adoption of sacubitril/valsartan for patients with heart failure with reduced ejection fraction: insights from Get With the Guidelines–Heart Failure (GWTG-HF). JACC: Heart Failure. 2017;5(4):305-9

^3^Ogden CL, Carroll MD, Lawman HG, Fryar CD, Kruszon-Moran D, Kit BK, et al. Trends in obesity prevalence among children and adolescents in the United States, 1988-1994 through 2013-2014. Jama. 2016;315(21):2292-9.

^4^Flegal KM, Kruszon-Moran D, Carroll MD, Fryar CD, Ogden CL. Trends in obesity among adults in the United States, 2005 to 2014. Jama. 2016;315(21):2284-91.

^5^Beckman AL, Herrin J, Nasir K, Desai NR, Spatz ES. Trends in cardiovascular health of US adults by income, 2005-2014. JAMA cardiology. 2017;2(7):814-6.

^6^Shah NS, Molsberry R, Rana JS, Sidney S, Capewell S, O’Flaherty M, et al. Heterogeneous trends in burden of heart disease mortality by subtypes in the United States, 1999-2018: observational analysis of vital statistics. BMJ. 2020;370.

**Comment 2: page 16 lines 286- The authors discussed how PCP densities mattered and any intervention for this point. However, according to Fig. 2, physician density may not have considerable effect, where IRR was 1.16 to 1.14 in age 65-84 and 1.67 to 1.57 in age 35-64. This means if PCP densities have completely been improved, IRR may be improved with the only improvement of 1.16 to 1.14. Is telemedicine worth trying, for example?**

Authors’ Response: We thank the reviewer for this comment, and we agree that physician density is not having considerable effect and we have revised our discussion to better emphasize this point. Specifically, in older adults, there is minimal change to the IRR. However, in younger adults, there does appear to be a dose dependent relationship between physician density and HF mortality as evidenced in Fig 3 (Fig 4 in the revised manuscript). Therefore, we have revised the text to modify the discussion regarding the role of physician density in HF-related mortality as well as the rural-urban disparities we observe in HF-related mortality.

- Page 15, lines 388-391: *“Our results do not identify a significant contribution of density of PCPs and cardiologists to county-level HF-related mortality. However, there appears to be a modest but significantly lower HF-related mortality rates with greater physician density among young rural adults with minimal to no effect in older rural adults when examined by quintiles (****Fig 4****).”*
- Page 15, lines 397-402: *“Interestingly, however, we found that the IRR between rural and urban counties was not significantly different when controlling for physician density continuously compared with the unadjusted model (Fig 3). Because changes in physician density act upstream in the causal pathway of HF-related mortality, changes in county-level HF-related mortality may not be manifested until several years after the corresponding change in physician density.”*
- Page 16, lines 415-417: *“It will be important for future studies to investigate local changes in HF-related mortality rates following these drastic shifts in access to health insurance, physicians, and hospital care, particularly among rural communities.”*

We also thank the reviewer for the important suggestion regarding the future role of telemedicine. We agree that telemedicine will play a key role in HF management going forward, particularly in the setting of the ongoing COVID-19 pandemic. We have emphasized this point in the text:

- Page 16, lines 418-421: *“Telemedicine may also be an effective way to mitigate the large differences in access to care between rural and urban patients and potentially reduce rural-urban disparities in HF prevention and management. This is of particular importance in the context of the current COVID-19 pandemic when telemedicine has become a more common mode of care delivery for chronic disease management.”*

**Comment 3: page 17 lines 320- "increase in HF-related mortality have been distributed unequally" but "universally in all race-sex subgroups". This does not mean the unequality is a reason for increase in HF-related mortality. In the same way as reviewer's comment #1, some explanation may be needed.**

Authors’ Response: Thank you for requesting this clarification. We did not intend to imply that inequality is a reason for increased HF mortality and have revised the language to better clarify this point. However, we sought to describe the heterogeneity within race-sex subgroups regarding the rate of increase in HF-related mortality.

We have further clarified the discussion section, which now reads:

- Page 17, lines 436-437:*“We also demonstrate that recent increases in HF-related mortality are different between race-sex subgroups.”*

**Comment 4: page 18 lines 325- Which results were the evidence of the number 45% and 33%?**

Authors’ Response: We have clarified the origin of these numbers. They reflect the change in mortality data that is shown in Table 1. The text now reads:

- Page 17, lines 441-445: *“Strikingly, between 2011 and 2018, HF-related mortality among young rural black men increased over 45% (AAMR 35.9 [31.8-40.0] per 100,000 population in 2011 versus 52.3 [47.4-57.3] in 2018) compared with a 33% increase in young rural white men (AAMR 15.6 [14.8-16.4] in 2011 versus 20.8 [19.8-21.7] in 2018). If current trends continue, HF-related AAMR among young rural black men will double by the year 2023 when compared with 2011.”*

-----------------------------------------------------------

**Reviewer #2: I confine my remarks to statistical aspects of this paper. Unfortunately, there was two major and several minor problems.**

Authors’ Response: We thank the reviewer for their time and thorough review of our work. We have responded in detail to the reviewer’s suggestions below.

**Comment 1: Since patients are clustered in counties, the assumption of independent errors is violated. The authors need to deal with this. One way is with a multilevel model.**

Authors’ Response: We thank the reviewer for bringing up this important point requesting clarification in the modeling strategy and data structure. For privacy reasons, patient-level data is not available from CDC WONDER. To perform the analysis to address our scientific question of trends and associations in county-level heart failure mortality, we queried CDC WONDER for aggregated county-level data in which each county was treated as an independent data point with a corresponding age-adjusted mortality rate based on prior publications examining county-level mortality trends. As such, the assumption of independent errors is not validated in our analysis. We respectfully agree this is an important point of clarification and have thus updated the methods to make this point clearer. The rationale for our analytic approach is based on the recommendations by the United States Centers for Disease Control (CDC) and National Vital Statistics System (NVSS) who curate and provide access to these nationwide data. Prior publications are now also included in our methods section identifying the rationale for the use of the county-level unit for mortality analyses.^1-4^ The text now reads:

- Page 5, lines 133-136: *“The rationale for our analytic approach is based on the recommendations by the United States Centers for Disease Control (CDC) and National Vital Statistics System (NVSS) who curate and provide access to these nationwide data and is consistent with prior CDC publications [27-30].”*

^1^Vaughan AS, Quick H, Schieb L, Kramer MR, Taylor HA, Casper M. Changing rate orders of race-gender heart disease death rates: An exploration of county-level race-gender disparities. SSM-population health. 2019;7:100334.

^2^Hall EW, Vaughan AS, Ritchey MD, Schieb L, Casper M. Stagnating National Declines in Stroke Mortality Mask Widespread County-Level Increases, 2010–2016. Stroke. 2019;50(12):3355-9.

^3^Vaughan AS, Ritchey MD, Hannan J, Kramer MR, Casper M. Widespread recent increases in county-level heart disease mortality across age groups. Annals of epidemiology. 2017;27(12):796-800.

^4^Vaughan AS, Schieb L, Casper M. Historic and recent trends in county-level coronary heart disease death rates by race, gender, and age group, United States, 1979-2017. PLoS One. 2020;15(7):e0235839.

**Comment #2: Counties are not simply rural or urban. First, the usual division is rural, suburban and urban. Second, some counties are a mix. I have seen data on the % urban, suburban and rural by congressional district (see Almanac of American Politics). If this isn't available by county, the authors should explain that. But e.g. Los Angeles County includes large areas that are pretty rural. (It's almost 5000 square miles).   (See also the comment below about age). At a minimum use the categories listed on lines 111-113 and don't group them.**

Authors’ Response: We thank the reviewer for this comment on rural-urban classification schemes for counties. The county is the smallest geographical unit of measurement available in CDC WONDER; data is unavailable by congressional district due to privacy concerns at a smaller geographic scale. However, we agree with the reviewer that there is significant heterogeneity at the county-level and have further emphasized this in the discussion as a key limitation. The text now reads:

- Page 18, lines 481-483: *“Counties spanning large geographical areas may be significantly heterogeneous with mixed rural and urban neighborhoods. Future dedicated analyses of differences in HF-related mortality over smaller geographical areas (i.e. congressional district) may provide additional insight.”*

Regarding the classification choices for our analysis: we chose the dichotomous classification of rural vs. urban to provide a meaningful grouping for the analysis based on prior publications^1-5^ and official recommendations on rural-urban classification from the National Center for Health Statistics (NCHS).

Our classification scheme is similar to the overall structure of the 2013 NCHS Classification Scheme for Counties, which initially designates counties as either metropolitain or nonmetropolitain (or urban versus rural, for the sake of clarity).^6^ Further subclassifications can be examined and do indeed show a graded association between rurality and HF-related mortality (Table below). However, based on the agreement with the official recommendations from the NCHS as well as being an established an accepted grouping in previous literature on rural-urban differences, we believe that the current rural-urban classification allows for the detection and characterization of meaningful-rural urban differences in HF-related mortality while also allowing for the identification of the finer points in an age- and race-sex specific analysis.

|  | AAMR 2011  (per 100,000 population) | AAMR 2018  (per 100,000 population) | AAPC |
| --- | --- | --- | --- |
| Noncore (most rural) | 67.8 (66.3-69.3) | 74.2 (72.7-75.7) | 1.6 (1.0-2.3) |
| Micropolitan | 63.3 (62.1-64.6) | 72.5 (71.2-73.8) | 2.2 (1.7-2.7) |
| Small metro | 60.6 (59.4-61.8) | 65.6 (64.4-66.8) | 1.4 (0.9-2.0) |
| Medium metro | 54.0 (53.2-54.8) | 60.7 (59.9-61.5) | 1.8 (1.4-2.3) |
| Large fringe metro | 47.8 (47.1-48.5) | 53.3 (52.6-54.0) | 1.8 (1.3-2.3) |
| Large central metro (most urban) | 50.2 (49.5-50.9) | 54.8 (54.1-55.5) | 1.6 (0.9-2.3) |

AAMR=age-adjusted mortality rate; AAPC=average annual percent change

^1^Shah NS, Molsberry R, Rana JS, Sidney S, Capewell S, O’Flaherty M, et al. Heterogeneous trends in burden of heart disease mortality by subtypes in the United States, 1999-2018: observational analysis of vital statistics. BMJ. 2020;370.

^2^Gong G, Phillips SG, Hudson C, Curti D, Philips BU. Higher US Rural Mortality Rates Linked to Socioeconomic Status, Physician Shortages, and Lack of Health Insurance. Health Affairs. 2019;38(12):2003-10.

^3^Matthews KA, Croft JB, Liu Y, Lu H, Kanny D, Wheaton AG, et al. Health-related behaviors by urban-rural county classification—United States, 2013. MMWR Surveillance Summaries. 2017;66(5):1.

^4^James CV, Moonesinghe R, Wilson-Frederick SM, Hall JE, Penman-Aguilar A, Bouye K. Racial/ethnic health disparities among rural adults—United States, 2012–2015. MMWR Surveillance Summaries. 2017;66(23):1.

^5^García MC. Potentially Excess Deaths from the Five Leading Causes of Death in Metropolitan and Nonmetropolitan Counties—United States, 2010–2017. MMWR Surveillance Summaries. 2019;68.

^6^Ingram DD, Franco SJ. 2013 NCHS urban-rural classification scheme for counties. 2014.

**Comment #3: Counties are problematic as a unit of analysis. Some counties have very few people and these counties will have very high variance. See Andrew Gelman's paper "All maps of parameter estimates are misleading".**

Authors’ Response: We thank the reviewer for bringing up this point, and we agree that this is an important limitation of the study. However, we respectfully note that these analyses are important for describing the heterogeneity in a country as diverse and large as the United States. We hope that analyses like these will be important to inform public health policies and lead to more in-depth analyses examining disparities at the neighborhood-level. We have added this to the limitations section, which now reads:

- Page 18, lines 483-485: *“Counties with low populations may have high variance in AAMRs. However, because counties with less than 20 HF-related mortality events were excluded from the study, the counties at the highest risk for significantly inflated or deflated AAMRs have been excluded.”*

**Comment #4: Don't categorize age. In *Regression Modelling Strategies* Frank Harrell lists 11 problems with categorizing continuous variables and sums up "Nothing could be more disastrous". Leave age in years and use a spline to assess nonlinearity**

Authors’ Response: This is an important point for clarification, and we thank the reviewer for requesting this clarification. Because we have already accounted for age in AAMRs, we did not want to over-adjust for this metric. Additionally, as with Comment #1 above, the data used in this study are not patient level data, but instead county-level data from which mortality rates are calculated. As a result, age of individual decedents is not available. Finally, grouping by age is a widely accepted practice when comparing differential epidemiological trends in AAMR between age groups. We have utilized these strategies in previous publications^1-3^ that have led to important insights into recent trends in cardiovascular disease epidemiology. For instance, in the present analysis, we utilize the same age groupings used in Glynn et al.^3^ based on our pre-planned analytic plan, where we described concerning trends in racial disparities in HF that differed between decedents 35-64 years versus 65-84 years. In addition, these age cutpoints are important in the United States given that 65 years is the age minimum to qualify for Medicare insurance coverage and makes these cutpoints more clinically meaningful. We excluded decedents less than 35 years to mitigate the contribution of congenital heart disease survivors towards heart failure mortality rates. For these reasons, we respectfully maintain that our current analysis strategy appropriately adjusts for age in mortality rate calculations and uses appropriate, widely accepted strategies for age categorization. We have revised the methods and discussion to clarify this rationale as well as note the limitations of this approach in the absence of patient-level data.

- Page 6, lines 160-161: *“Age was categorized as 35-64 years and 65-84 years based on age of 65 years being required for Medicare eligibility and previously published analyses [5, 10, 14].”*
- Page 18, lines 472-473:*“As such, we could not include the age of individual decedents in our regression modeling strategies.”*

^1^Shah NS, Lloyd-Jones DM, O’Flaherty M, Capewell S, Kershaw K, Carnethon M, et al. Trends in cardiometabolic mortality in the United States, 1999-2017. JAMA. 2019;322(8):780-2.

^2^Shah NS, Molsberry R, Rana JS, Sidney S, Capewell S, O’Flaherty M, et al. Heterogeneous trends in burden of heart disease mortality by subtypes in the United States, 1999-2018: observational analysis of vital statistics. BMJ. 2020;370.

^3^Glynn P, Lloyd-Jones DM, Feinstein MJ, Carnethon M, Khan SS. Disparities in Cardiovascular Mortality Related to Heart Failure in the United States. Journal of the American College of Cardiology. 2019;73(18):2354.

**Comment #5: Line 98 What about people who are neither Black nor White? What about Latinx people, who can be of any race?**

Authors’ Response: We thank the reviewer for raising this important point about race and ethnicity in this analysis. We agree that consideration of HF-related mortality among other racial and ethnic subgroups is an important epidemiological question that needs to be addressed. Regarding our study, we did not utilize ethnicity due to concerns regarding its accuracy on death certificates in the United States. According to the CDC WONDER website, “Hispanic origin was not reported on the death certificate for some deaths” during the study period.

Additionally, the CDC clarifies that “Information included on the death certificate about the race and Hispanic ethnicity of the decedent is reported by the funeral director as provided by an informant, often the surviving next of kin, or, in the absence of an informant, on the basis of observation. Race and ethnicity information from the census is by self-report. To the extent that race and Hispanic origin are inconsistent between these two data sources, death rates will be biased.”

Conversely, Arias et al. demonstrated that racial and ethnic identification for White and Black populations are excellent. For these reasons, we limited our analysis to White and Black decedents over the study period. We have clarified this in the methods section, which now reads:

- Page 6, lines 165-169: *“Due to concerns over the reliability of accurate reporting of ethnicity (i.e. Hispanic or Latinx) [15] on death certificate data, we stratified only by race (Black or White), for which death certificates have been shown to be exceedingly accurate [34]. Too few HF-related mortality events precluded the inclusion of other race and ethnicity groups (e.g. Native American, Asian, or Pacific Islander).”*

^1^Glynn P, Lloyd-Jones DM, Feinstein MJ, Carnethon M, Khan SS. Disparities in Cardiovascular Mortality Related to Heart Failure in the United States. Journal of the American College of Cardiology. 2019;73(18):2354.

^2^Shah NS, Lloyd-Jones DM, O’Flaherty M, Capewell S, Kershaw K, Carnethon M, et al. Trends in cardiometabolic mortality in the United States, 1999-2017. JAMA. 2019;322(8):780-2

-----------------------------------------------------------

**Reviewer #3: Pierce and colleagues present an analysis of HF related mortality rates in rural versus urban counties and describe well disparities and factors associated with disparities. The manuscript addresses an important question and is impactful and well written. The question is relevant from a public health perspective and important in considering the heart failure epidemic as it evolves.**

Authors’ Response: We thank the reviewer for their time and appreciate the overall enthusiasm regarding our work. We have responded in detail to the reviewer’s suggestions below.

**Comment #1: The authors describe linkage of several databases for their study; is there data as to the veracity of the linkage and has this data linkage been used in prior studies? Such information and citations would be helpful.**

Response: We thank the reviewer for this query. The analytic approach and databases linked in our study were largely based on several recent papers using similar data sources and analytic strategy.^1-5^ We have amended the manuscript with this citation, which now reads:

- Page 5, lines 127-133: *“Similar to previous studies [16-20], data on county-level factors were obtained from the US Census Bureau Population and Housing Unit Estimates [21], Small Area Income and Poverty Estimates Program [22], Small Area Health Insurance Estimates Program files [23]; US Bureau of Labor Statistics Local Area Unemployment Statistics file [24]; Centers for Disease Control and Prevention Behavioral Risk Factor Surveillance System [25]; and the Health Resources and Services Administration Area Health Resources File [26] and subsequently linked using county identifiers to the CDC WONDER mortality data.”*

^1^Khatana SAM, Bhatla A, Nathan AS, Giri J, Shen C, Kazi DS, et al. Association of medicaid expansion with cardiovascular mortality. JAMA Cardiology. 2019;4(7):671-9.

^2^Hong Y-R, Mainous AG. Development and Validation of a County-Level Social Determinants of Health Risk Assessment Tool for Cardiovascular Disease. The Annals of Family Medicine. 2020;18(4):318-25.

^3^Langabeer JR, Chambers KA, Cardenas-Turanzas M, Champagne-Langabeer T. County-level factors underlying opioid mortality in the United States. Substance abuse. 2020:1-7.

^4^Mac McCullough J, Leider JP. Associations between county wealth, health and social services spending, and health outcomes. American journal of preventive medicine. 2017;53(5):592-8.

^5^Song S, Trisolini MG, LaBresh KA, Smith SC, Jin Y, Zheng Z-J. Factors Associated With County-Level Variation in Premature Mortality Due to Noncommunicable Chronic Disease in the United States, 1999-2017. JAMA Network Open. 2020;3(2):e200241-e.

**Comment #2: Along similar lines, the authors describe use of ICD10 code in the multiple cause of death position with CVD as the primary cause; is such an approach validated to identify heart failure deaths? If so, what is the sensitivity and specificity of this approach?**

Authors’ Response: We thank the reviewer for requesting this clarification. Similar to Comment #1, this definition was used to maintain consistency with prior publications in the field.^1-3^ However, we agree that use of administrative coding is always subject to limitations and may be subject to miscoding. Therefore, we have further highlighted this in our limitations as well. However, these data provide the most robust assessment of national burden of cardiovascular mortality related to heart failure. We have revised the manuscript to reflect our decision-making process, which now reads:

- Pages 6, lines 154-157*: “Therefore, to capture the broad burden of cardiovascular mortality related to HF, we included decedents in which HF (defined as International Classification of Diseases, 10th Revision [ICD-10] code I50) was listed as a multiple cause of death with CVD (I00-I78) listed as the underlying cause of death similar to previous studies [10, 14, 23].”*
- Pages 18-19, lines 490-496: *“Finally, mortality rates in this study are based on death certificate data and ICD-10 codes, which is subject to miscoding and does not distinguish between HF phenotypes such as HF with reduced or preserved ejection fraction. Instead, this definition encompasses a comprehensive burden of overall HF-related mortality.”*

^1^Glynn P, Lloyd-Jones DM, Feinstein MJ, Carnethon M, Khan SS. Disparities in Cardiovascular Mortality Related to Heart Failure in the United States. Journal of the American College of Cardiology. 2019;73(18):2354.

^2^Sidney S, Go AS, Jaffe MG, Solomon MD, Ambrosy AP, Rana JS. Association between aging of the US population and heart disease mortality from 2011 to 2017. JAMA Cardiology. 2019;4(12):1280-6.

^3^Shah NS, Molsberry R, Rana JS, Sidney S, Capewell S, O’Flaherty M, et al. Heterogeneous trends in burden of heart disease mortality by subtypes in the United States, 1999-2018: observational analysis of vital statistics. BMJ. 2020;370.

**Comment #3: The authors chose to exclude those aged 85 years and older and provide rationale; how do they feel this choice could bias the results? Could be discussed in the limitation section**

Authors’ Response: We thank the reviewer for raising this important point, and we agree that the exclusion of decedents aged 85 years or older may introduce bias into the AAMR estimates. However, we excluded these decedents based on studies demonstrating poor accuracy of death certificate data among persons in this age group, particularly with regard to HF diagnoses. Therefore, we excluded these decedents from the study to reduce the likelihood of type 1 error, knowing that this potentially biases us towards the null hypothesis. We believe this analysis strategy likely leads to conservative estimates of HF-related mortality, but that the annual trends and between-group differences in AAMRs are likely more accurate. We appreciate this recommendation and have further clarified this in the limitations section.

- Page 18, lines 486-490: *“Decedents aged 85 years or older were excluded from the study given concerns related to accuracy of death certificate coding in this age range [32]. While this introduces potential bias, excluding decedents aged 85 years or older likely resulted in lower, conservative estimates of HF-related mortality, but was unlikely to significantly affect annual trends and between-group differences in AAMRs.”*

**Comment #4: The authors could consider addition of interaction terms in the model to quantify the interaction of time and rural county with IRR**

Authors’ Response: We would like to thank the reviewer for this important suggestion. The first goal of our analysis was to examine trends in heart failure mortality in rural and urban counties. The second goal was to examine the relationship between county-level factors and differences in heart failure mortality between rural and urban counties over the entire study period between 2011-2018 to avoid drop-out from smaller counties if a per-annual analysis were to be performed. Therefore, in this second part of the pre-specified analysis, we did not account for time. However, our sensitivity analysis using the most recent data for county-level factors (**S4 Table**) demonstrates little change in rural versus urban incidence rate ratios using compared with models using baseline county-level factors, suggesting there was little variation with time over the study period.

**Comment #5: The statistical methods would benefit from a discussion about why the negative binomial model was chosen rather than poisson model and description of how model assumptions were assessed**

Authors’ Response: We thank the reviewer for raising this point of clarification for our decision-making regarding regression modeling. We have clarified in the methods section:

- Page 8, lines 205-206*: “Due to overdispersion of the data used in our regression analyses, we utilized negative binomial regression rather than Poisson regression.”*

**Comment #6: The statistical section would also benefit from a brief description of how the authors chose the covariates for each model- based on conceptual framework, known confounders, etc?**

Authors’ Response: We thank the reviewer for raising this question regarding the modeling strategies used in our analysis and agree this should be clarified early in the methods. Briefly, our modeling strategy was designed to provide insight into the association of both broad categories of covariates (i.e. demographic characteristics, socioeconomic characteristics, risk factor prevalence, and physician density) and individual county-level covariates (e.g. percentage of county residents who are uninsured) to rural-urban disparities in HF-related mortality. Model variables were included based on known association with HF and cardiovascular disease morbidity and mortality with reliable publicly available county-level data and based on prior publications in county-level analyses of heart disease. We have included this rationale in the methods section:

- Page 8, lines 217-224: *“Our modeling strategy was designed to provide insight into the association of both broad categories of covariates (i.e. demographic characteristics, socioeconomic characteristics, risk factor prevalence, and physician density) and individual county-level covariates (e.g. percentage of county residents who are uninsured) to rural-urban disparities in HF-related mortality. Individual county-level covariates were chosen based upon known association with HF and cardiovascular disease morbidity and mortality with reliable, publicly available county-level data and consistent with prior publications examining differences in county-level mortality broadly in heart disease (16).”*

**Comment #7: What was the rationale for choice of the study time period?**

Authors’ Response: We agree with the reviewer that it is important to clarify the reasoning for the chosen study period. We have clarified in the methods section:

- Page 5-6, lines 143-152: *“The study period began at the well-described inflection point in 2011 at which point HF-related mortality in the United States began to increase [4-6] and extends through the most recent year for which data are publicly available in CDC WONDER, 2018.”*

**Comment #8: I might include in the manuscript the Figure equivalent of Table 1 which would be impactful (Figure S1) and also include the trends stratified by age and race in the figure**

Response: We thank the reviewer for this suggestion and have included **S1 Fig** as the new **Fig 1** in the manuscript.
